# Frequency of Pulmonary Hypertension in Hemodialysis Patients

**DOI:** 10.12669/pjms.306.5525

**Published:** 2014

**Authors:** Kunwer Naveed Mukhtar, Syed Mohkumuddin, Sumbal Nasir Mahmood

**Affiliations:** 1 Dr. Kunwer Naveed Mukhtar, MD, DABN, Associate Professor, Head Department of Nephrology & Transplantation, Liaquat National Hospital & Medical College, Karachi, Pakistan.; 2 Dr. Syed Mohkumuddin, MBBS, FCPS, Consultant Nephrologist, Department of Nephrology, Sandamen Provincial Hospital Quetta, Pakistan.; 3 Dr. Sumbal Nasir Mahmood, MD, DABN, Associate Professor, Department of Nephrology, Dr. Ziauddin Hospital & Medical College, Karachi, Pakistan.

**Keywords:** ESRD = End stage renal disease, PH = Pulmonary hypertension, HD = Hemodialysis, CKD = Chronic kidney disease, PAP = Pulmonary artery pressure, SPAP = Systolic pulmonary arterial pressure

## Abstract

***Objective: ***Pulmonary hypertension (PH) has been described in hemodialysis (HD) patients and has been associated with increased morbidity and mortality. Our objective was to determine the prevalence of pulmonary hypertension in patients on regular hemodialysis.

***Methods: ***This cross sectional study was conducted in Department of Nephrology, Liaquat National Hospital Karachi from April 2013 to March 2014. Eighty patients of end stage renal disease (ESRD), on maintenance hemodialysis (HD); underwent Trans thoracic Echocardiography were selected. Systolic pulmonary arterial pressure (SPAP) was recorded. Pulmonary hypertension was defined as, pulmonary artery pressure (PAP) greater than 30 mm Hg at rest. Pulmonary hypertension was further divided into mild (PAP b/w 30-45mmHg), moderate (PAP b/w 45-65mmHg) and severe pulmonary hypertension (PAP > 65mmHg). The effect of different vascular accesses, age, gender, dialysis vintage on the development of pulmonary hypertension was observed.

***Results: ***Out of 80 patients, 45 patients (56%) had pulmonary hypertension (PH); 25(55.5%) had moderate, 13(29%) had mild, and 7 (15.5%) patients had severe pulmonary hypertension (PH). Pulmonary hypertension was present in 41(60%) patients with AVF, 3(27%) patients with tunnel cuffed catheter and 1 patient had AV bridge graft. Pulmonary hypertension was more common in females; present in 28 females (67%) and 17 males (45%), that was statistically significant (p<0.05). Mean duration of hemodialysis in (months) of patients with PH was 20.93 ± 12 vs. 10.29 ±10 in patients without PH (p<0.05). Age had no relation to development of PH.

***Conclusion: ***ESRD patients on HD have strong tendency to develop PH. Our study demonstrated that PH is more common among females. Duration of hemodialysis and AV access has strong relation to the development of PH.

## INTRODUCTION

Pulmonary hypertension (PH) is defined as systolic pulmonary arterial pressure (SPAP) greater than 30 mm Hg at rest determined by Doppler echocardiography.^   1 ^ Chronic kidney disease (CKD) is a challenging issue for health care providers and major burden for health care. Cardiovascular disease is a well-recognized and important source of mortality in patients with chronic kidney diseas.^2^^-^^4^ It accounts for approximately 50 percent of deaths in dialysis patients.^5^ Aside from coronary artery disease, other forms of cardiovascular disease are also prevalent in chronic kidney disease. Pulmonary hypertension (PH) has been described in hemodialysis patients.^6^^,^^7^  It is a progressive disorder with increased morbidity and mortality.^8^ Yigle et al. in the study reported a significantly lower survival rate in HD patients with PH with their counterparts without PH.^9^

The clinical manifestations of secondary PH are frequently masked by the underlying etiology and the diagnosis may be confirmed only after the onset of right ventricular failure. Echocardiography has enabled non invasive accurate estimation of pulmonary arterial hypertension.^10^ Pulmonary hypertension in ESRD patients may be multifactorial.^11^^,^^12^

Chronic volume overload, metabolic derangements affecting pulmonary vasculature, alterations in calcium and phosphate metabolism causing metastatic pulmonary artery calcification, chronically increased blood flow from arteriovenous fistula or arteriovenous graft, all these may predispose to elevated pulmonary pressures. The problem is usually overlooked and under addressed which ultimately leads to irreversible heart failure and death. We conducted a study to determine the prevalence of pulmonary hypertension in patients on maintenance hemodialysis irrespective of angioaccess so as to emphasize the importance of early and timely detection of pulmonary hypertension by doing regular screening echocardiography. Those found to have pulmonary hypertension, can be offered alternative method of dialysis, reversal of AV fistula or may be suggested to have early renal transplant. Adapting these measures can reverse pulmonary hypertension.

## METHODS

This cross sectional study was conducted in Department of Nephrology, Liaquat National Hospital, Karachi from April 2013 to March 2014. Patients were enrolled if they gave written consent for participation. Approval was obtained from hospital ethical committee. We studied 80 patients of End stage renal disease (42 female and 38 male) on regular HD via permanent AV accesses. These patients were dialyzed three times a week, each session lasting for four hours. Patients with chronic obstructive lung disease, chest wall or parenchymal lung disease, previous pulmonary embolism, collagen vascular disease, moderate or severe mitral or aortic valve disease and having obstructive sleep apnea were excluded. All patients underwent Tran thoracic Echocardiography by a cardiologist. Echocardiography was performed post dialysis when patients were at optimal dry weight. Systolic pulmonary artery pressure was measured. Ejection fraction was also estimated. Effect of different vascular accesses, age, gender, dialysis vintage on the development of PH were observed.

***Statistical Analysis: ***Data was collected on pre designed Performa and analyzed using SPSS version 22. Chi square test was used for estimating the occurrence of categorical variables and p<0.05 was considered significant. Regression analysis was used to analyze effect of duration of hemodialysis on PH. Student t test was also used to compare difference in means of various parameters.

## RESULTS

Results are shown in Table-I. Out of total 80 patients enrolled in the study, 42 patients (52.5%) were female and 38 patients (47.5%) were male. Minimum duration of hemodialysis was two month and maximum was 60 months and mean duration in months was 16.28 ± 12.2. Out of 80 patients enrolled, 68 patients (85%) were having AVF, 11 Patients (13.8%) had tunneled cuff catheter and 1 patient (1.3%) had AV bridge graft. In our study, PH was present in 45 patients (56%). The mean value for PAP (mmHg) was 38.5 ± 19.17. Patient with minimum age having PH was of 18 year and of maximum age was 72 year. Mean age in (year) for patients with PH was 53.44 ± 12.2, and without PH was 53.86 ± 13.5.

**Fig.1:**
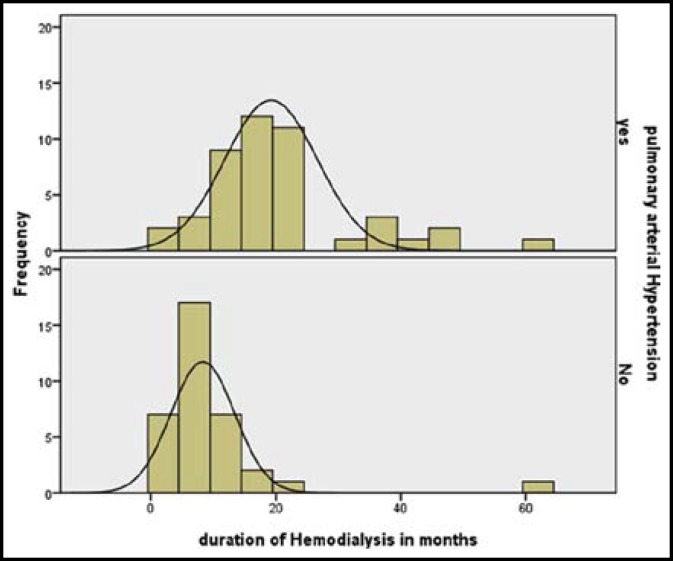
Impact of Hemodialysis duration on PH.

**Fig.2:**
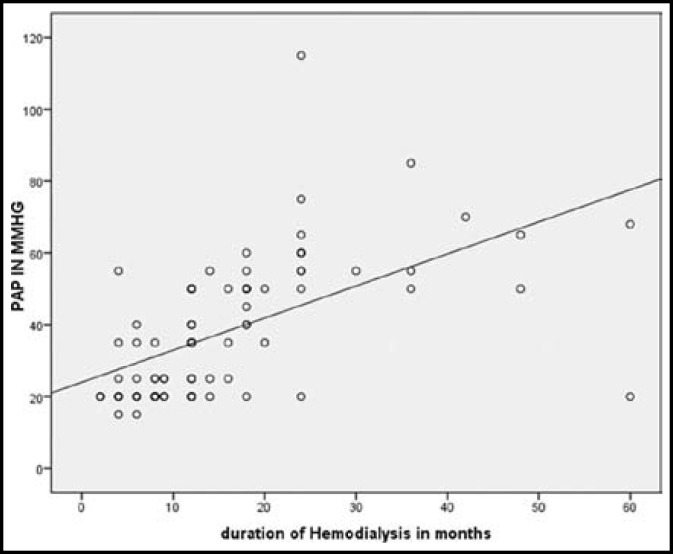
Impact of Hemodialysis duration on severity of PH.

Among 45 patients with PH, 11 patients (27.5%) were 60 years old. PH was more common in females, present in 28 females (67%) and 17 males (45%). Odd ratio (OR= 0.405, 95% CI= 0.164 –1.001, P=0.048). 13 patients (29%) were found to have mild PH, 25 patients (55.5%) had moderate PH and 7 patients (15.5%) had severe PH. Among AVF group, PH was present in 41/68 (60%) patients, in tunneled cuff catheter group, PH was present in 3/11(27%) patients. Using Independent Sample T test (t=2.65, df=19.1 and P=0.015), PH had significant relation to AVF. For patients with PH, the mean value for duration on hemodialysis (in months) was 20.93 ± 12 and those without PH was 10.29 ± 10. Fig.1 shows, PH found more in patients below 20 month of duration on dialysis. The effect of duration of HD on PH is shown in Fig.2. Regression analysis shows (r^2^=0.324, p=<0.001) i.e. 32% of variance in PH is explained by duration of hemodialysis. More the duration on hemodialysis, more severe the PH was. 

## DISCUSSION

Chronic diseases like hypertension, diabetes mellitus are continuously on rise. These disease are the leading causes of end stage renal disease. Cardiovascular disease accounts for more than 50% of deaths among patients with ESRD.^13^ Certain factors have been proposed to contribute to this exceptionally increased risk, including dyslipidemia, homocysteinemia, oxidative stress of uremia and hemodialysis in this population. Role of hyperphosphatemia, AVF, elevated levels of the calcium-phosphorus product and hyperparathyroidism in the development of cardiovascular disease in ESRD has been evaluated.^14^ Once ESRD develops, the patient will need either renal transplant or dialysis. Dialysis is a double edge sword, beside its very vital role as renal replacement therapy; it has very serious long term effects. One of these is newly recognized disorder of PH in patients with ESRD. It is the most under addressed complication associated with high mortality and morbidity.^8^

**Table-I:** Characteristics of patients with and without PH

**Characteristics**		**Pulmonary Hypertension**		**P- Value**
		Yes (45)	No (35)	
Age (years)		53.44±12.2	53.86±13.5	0.22
Male / Female		17/28	21/14	0.048(<0.05)
Duration of dialysis (months)		20.93±12	10.29±10	<0.001
AV access	Fistula(68)	41 (60%)	27 (40%)	0.015(<0.05)

The prevalence of PH has been reported to be between 25%-51% in various studies.^14^^-^^16^ The highest incidence of about 58.6% was reported by Fabio Fabbian et al.^16^ However, most of these studies were retrospective and based on patients undergoing echocardiography for clinical indications hence had pre-selection bias. Our study, to the best of our knowledge, is the first one to address this important issue in this part of the world. The results of our study were also consistent with International data and we found 56% prevalence of PH in HD patients.

We also looked at factors that could be contributing to the development of PH in this population. Among these was vascular access for HD. Arteriovenous shunts created for hemodialysis have been identified as cause of unexplained PH in patients with end-stage renal disease. Yigla et al.^6^ in their study of 58 patients with CRF receiving long-term hemodialysis via arteriovenous access found PH in 39.7% of patients. Another study by Mordechai Yigla^17^ in 12 CKD patients found 42% of patients without PH increased their systolic PAP values by more than 10 mm Hg following AV access formation. Although these studies were small, however they suggest that AV access formation is a risk factor for PH.

Our study supported what has been found in the above studies. We found significant relationship of PH and AVF with 60% of patients being dialyzed through AVF had PH (p=0.05). We also studied the effect of various other hemodialysis access on the development of PH including patients with permanent catheters and AV bridge graft. Although our tunneled cuff catheter group was small and could not be followed for progression of PH for longer time however they were also noted to have a 27% prevalence of PH which was not statistically significant.

Gender has also been implicated as an independent risk factor for PH. Various studies have demonstrated that PH is detected more frequently in women than in men. Mona Amin et al.^18^ reported a higher prevalence of PH, 48% in women. Havlucu et al.^7^ studied 25 patients, female to male ratio of patients with PH was 60% vs. 40%. Our study also found higher prevalence in females, 52% compared to 48% in males, that was statistically significant. Similarly, we could not find a strong correlation between increasing age and development of PH.

Duration on hemodialysis (dialysis vintage) has direct relationship to the development of PH.^19^ We also found the same relationship of PH with the dialysis vintage. In addition, it was observed that patients who were on HD for 30 months or above, moderate PH was present in 3 patients and severe in 4 patients, hence dialysis duration can be associated with increased severity of PH, however longer studies and more number of patients are required to validate this observation.

An important finding in our study was that more number of cases of PH were found in our patients who were below 20 month of duration on hemodialysis. For patients with PH, the mean value for duration on hemodialysis (in months) was 20.93 ± 12 This is in contrast to other studies where it was 60 ± 36 months by Mona Amin.^11^ In study by Fabio Fabbian et al.,^16^ it was 40 ± 48 months. We do not know the exact etiology of early onset of PH but in our patients it could be probably the result of late onset of hemodialysis. Most of our patients do not start hemodialysis till they are uremic and although have been staged as ESRD, they delay it due to financial, socioeconomic, cultural and low literacy rate reasons. The early onset of PH is however a challenging issue, because if it progresses in the same fashion would lead to fatal consequences and irreversible right sided heart failure resulting in increased morbidity and mortality. Further studies are needed to find the exact etiology and cause of early onset of PH in our population so that proper measures may be taken.

Our study has some limitations. First of all, we did not have baseline transthoracic echocardiograms on our patients and we do not know if patients had PH prior to initiating HD. Also, we could not correlate the true risk of HD duration on development and severity of PH, as well as duration of AV access prior to starting HD.

## CONCLUSION

We conclude that PH is a significant problem in ESRD patients undergoing HD that needs to be addressed in a timely manner in order to avoid high risk of morbidity and mortality.
